# Epigenome-wide association study in healthy individuals identifies significant associations with DNA methylation and PBMC extract VEGF-A concentration

**DOI:** 10.1186/s13148-020-00874-w

**Published:** 2020-06-05

**Authors:** Vesna Gorenjak, Dwaine R. Vance, Sébastien Dade, Maria G. Stathopoulou, Lauren Doherty, Ting Xie, Helena Murray, Christine Masson, John Lamont, Peter Fitzgerald, Sophie Visvikis-Siest

**Affiliations:** 1 IGE-PCV, Inserm, Université de Lorraine, F-54000 Nancy, France; 2 Randox Laboratories Limited, Crumlin, Co. Antrim, Northern Ireland UK; 3 Department of Internal Medicine and Geriatrics, CHU Technopôle Nancy-Brabois, Rue du Morvan, F-54511, Vandoeuvre-lès-, Nancy, France; 4 INSERM UMR U1122, IGE-PCV, Faculté de Pharmacie—Université de Lorraine, 30 rue Lionnois, 54000 Nancy, France

**Keywords:** EWAS, VEGF, Methylation, Epigenetics

## Abstract

**Introduction:**

Vascular endothelial growth factor A (VEGF-A) is a chemokine that induces proliferation and migration of vascular endothelial cells and is essential for both physiological and pathological angiogenesis. It is known for its high heritability (> 60%) and involvement in most common morbidities, which makes it a potentially interesting biomarker. Large GWAS studies have already assessed polymorphisms related to VEGF-A. However, no previous research has provided epigenome-wide insight in regulation of VEGF-A.

**Methods:**

VEGF-A concentrations of healthy participants from the STANISLAS Family Study (*n* = 201) were comprehensively assessed for association with DNA methylation. Genome-wide DNA methylation profiles were determined in whole blood DNA using the 450K Infinium BeadChip Array (Illumina). VEGF-A concentration in PBMC extracts was detected using a high-sensitivity multiplex Cytokine Array (Randox Laboratories, UK).

**Results:**

Epigenome-wide association analysis identified 41 methylation sites significantly associated with VEGF-A concentrations derived from PBMC extracts. Twenty CpG sites within 13 chromosomes reached Holm-Bonferroni significance. Significant values ranged from *P* = 1.08 × 10^−7^ to *P* = 5.64 × 10^−15^.

**Conclusion:**

This study exposed twenty significant CpG sites linking DNA methylation to VEGF-A concentration. Methylation detected in promoter regions, such as TPX2 and HAS-1, could explain previously reported associations with the *VEGFA* gene. Methylation may also help in the understanding of the regulatory mechanisms of other genes located in the vicinity of detected CpG sites.

## Background

Recent developments and discoveries in epigenetics provided new insights into disease regulation, among which exploration of DNA methylation has become the most intriguing [1]. DNA methylation forms 5-methylcytosine on the CpG (cytosine-phosphate-guanine) site of a genome and normally results in silencing of the gene that is encoded in the sequence [2]. This particularity was researched in various epigenome-wide methylation studies (EWAS), which managed to relate individual CpGs with cardiovascular diseases [3], cancer [4] and other pathologies [5, 6]. In some cases, CpGs significantly associated with a certain disease are found on genes known to be involved with the aforementioned pathology or in promoter regions controlling gene expression [7]. In many cases, associations with chromosomal positions of methylated sites and disease are not obvious. Intergenic regions with CpG islands are thus systematically studied to elucidate the role of methylation in genomic regions distant from protein-coding regions [8].

Vascular endothelial growth factor A (VEGF-A) is a myogenic protein that induces angiogenesis, endothelial cell proliferation and plays an important role in the regulation of vasculogenesis [9]. VEGF-A is involved in the pathogenesis of cardiovascular disease [10], as well as other chronic diseases such as cancer [11], type 2 diabetes [12], osteoporosis, osteoarthritis [13] and chronic obstructive pulmonary disease (COPD) [14]. Anti-VEGF medications containing humanized antibody that blocks angiogenesis by inhibiting VEGF-A have already entered the market to treat a number of cancers, such as colon cancer, lung cancer, glioblastoma and renal cell carcinoma, as well as age-related macular degeneration [15–17].

The involvement of VEGF-A in various diseases makes it a universal biomarker with great potential for patient stratification in personalized medicine. The precise understanding of its biological and genetic regulation is required to fully appreciate its clinical potential. In previous years, a major effort has resulted in the discovery of several genetic variants with strong effects on growth factors, in particular VEGF-A concentration, using well-powered genome-wide association studies (GWAS). Ten genome-wide significant VEGF-A-associated SNPs [18, 19] that explained more than 50% of its individual variability have been identified. VEGF-A concentration is highly heritable reaching > 60% as demonstrated in the STANISLAS Family Study (SFS) [20]. Previous research has not yet investigated the role of epigenetics, such as DNA methylation on VEGF-A concentration. Therefore, epigenetic regulation could explain the missing heritability components [21].

Epigenetics is the study of gene transcription, regulation and expression that are not directly caused by the alteration of the genomic DNA sequence. DNA methylation occurs mostly on cytosine residues positioned in CpG islands (high density of CG dinucleotides) within a promoter region, transcription start site (TSS), first or second exons of a gene, in an enhancer region, or upstream from genes with CpG island shores (2 kb) or CpG shelves (2–4 kb) [2]. Previous studies have shown that epigenetics plays an important role in the regulation of promoter regions of *VEGFA* [22, 23] and *VEGFR* genes [24, 25], but no previous research studies have performed an EWAS of VEGF-A concentration to determine the methylation sites responsible for the regulation of *VEGFA*. As VEGF-A plays a distinct role in the development of several chronic diseases, the discovery of its epigenetic regulation mechanisms may contribute to a better understanding of these disorders and contribute in the research of new therapeutic possibilities.

To this end, we performed an EWAS on VEGF-A concentrations, measured from PBMC extracts in a healthy population, in order to identify possible epigenetic mechanisms involved in VEGF-A regulation before the pathological onset of chronic disease. We performed a large in silico analysis to detect possible repeating patterns of CpG chromosomal positions that could explain the role of each individual CpG site in *VEGFA* regulation.

## Results

In this investigation, we set out to explore links between genome-wide DNA methylation and PBMC extract VEGF-A levels, in a population of 201 healthy individuals from the SFS. The characteristics of the studied population are presented in Table 1. Genome-wide methylation profiling of bisulfite-converted genomic DNA was performed by Illumina HumanMethylation450 bead array (Illumina Inc., San Diego, CA, USA).

**Table 1:** Population characteristics

	Mean	SD	Median [interquartile range]
Age (years)	28.3	14.8	33.8 [13.25–42.08]
Sex (male %)	50.2	-	-
VEGF-A (pg/mL)	59.3	75.5	43.4 [23.67–66.45]
BMI (kg/m^2^)	21.6	4.0	21.3 [18.58–24.41]
Neutrophils (10^8^/l)	53.77	9.14	53.6 [47.5–60.6]
Lymphocytes (10^8^/l)	36.01	8.46	36.4 [29.7–41.2]
Monocytes (10^8^/l)	6.22	2.45	5.6 [4.6–7.4]
Eosinophils (10^8^/l)	2.84	1.98	2.2 [1.4–3.8]
Basophils (10^8^/l)	0.64	0.39	0.6 [0.4–0.9]

*SD* standard deviation, *VEGF*-*A* vascular endothelial growth factor A, *BMI* body mass index. Neutrophils, lymphocytes, monocytes, eosinophils and basophils represent mean individual blood cell counts of studied population

**Fig. 1:**
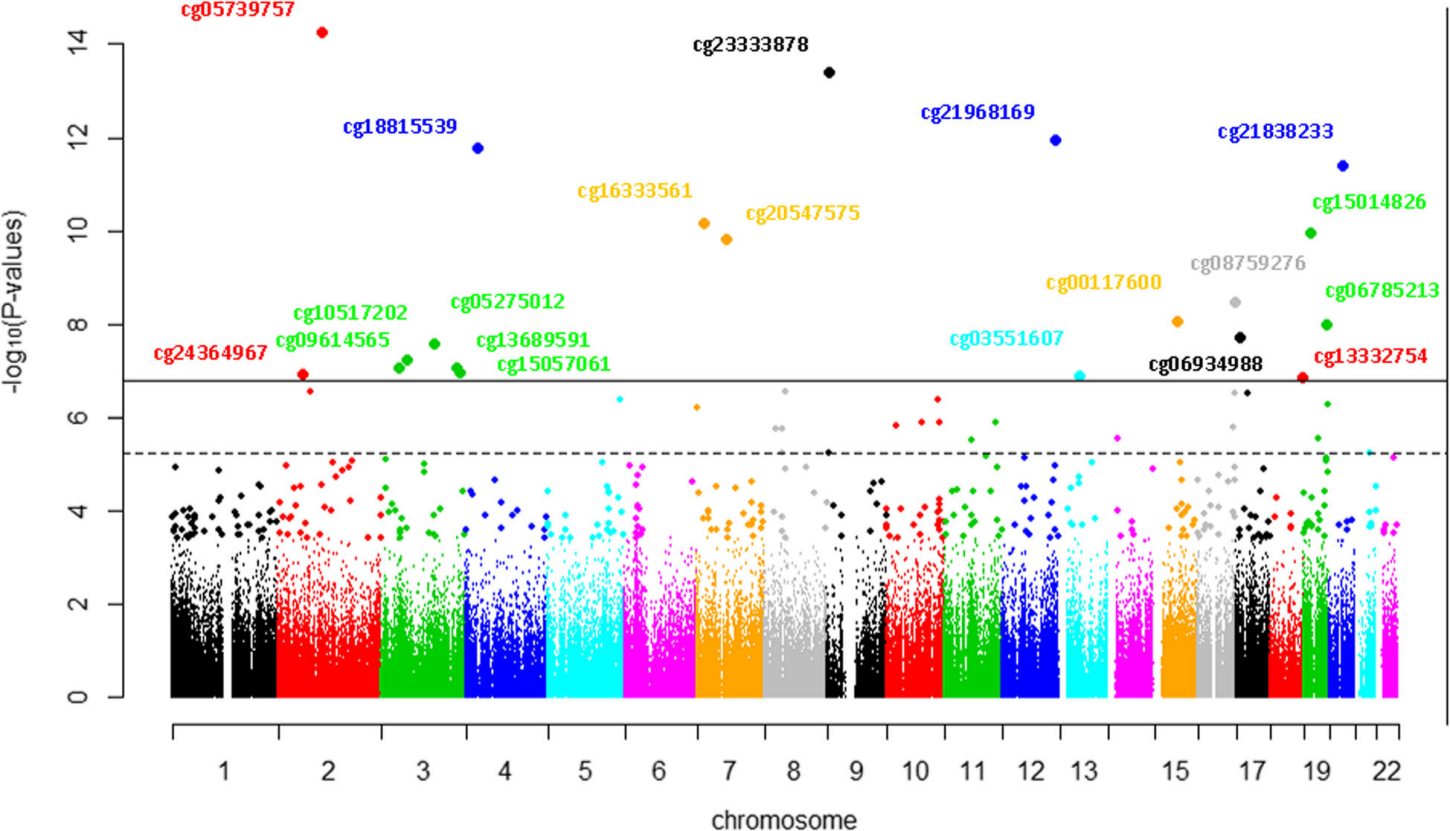
Manhattan plot displaying adjusted *P* values of the association between methylation probes and VEGF-A concentration in cell extracts. The dotted line represents FDR value, and points above the full line indicate results that were significant after Holm-Bonferroni testing

**Fig. 2:**
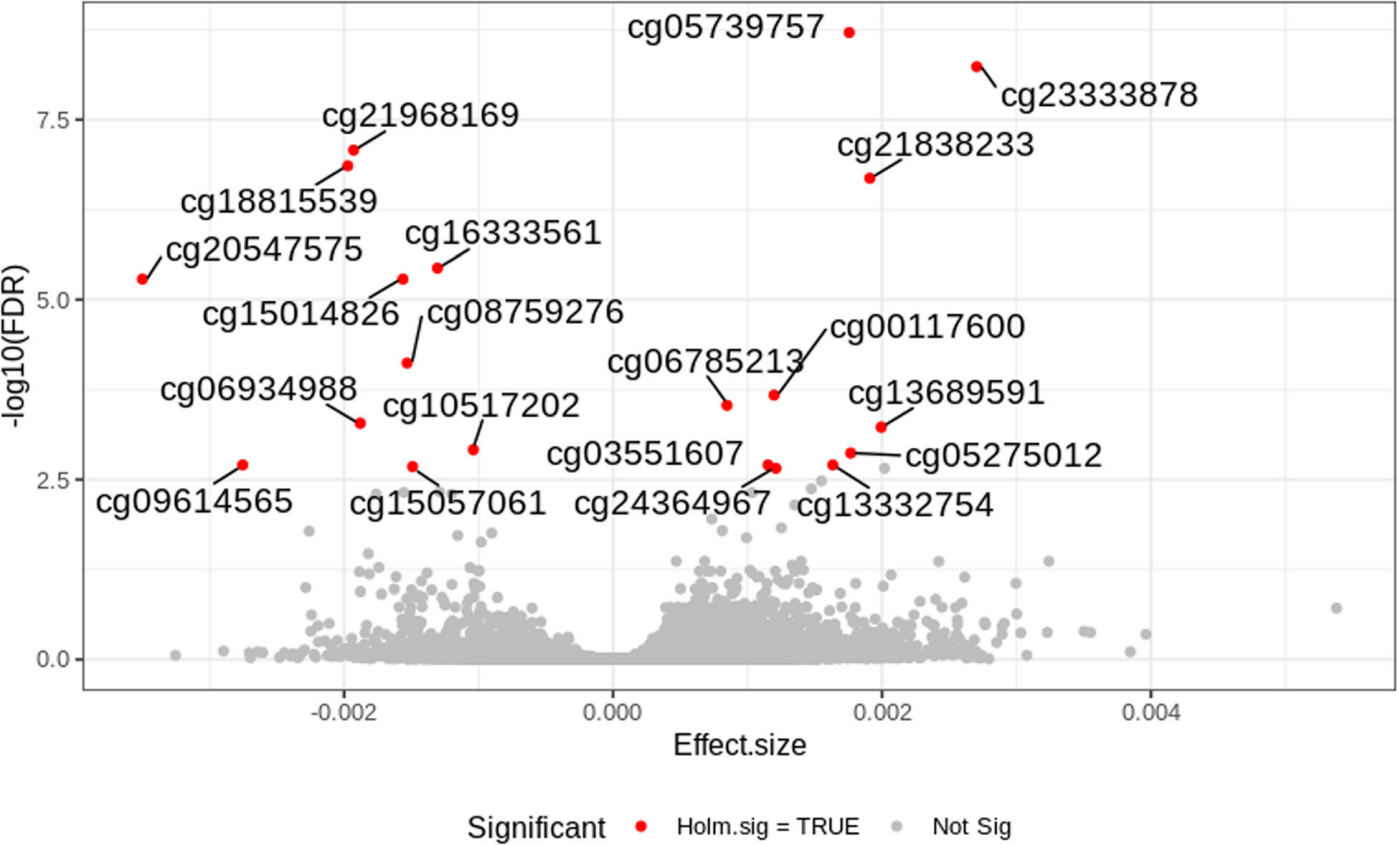
Volcano plot showing the direction of all associations between DNA methylation and VEGF-A. CpG sites passing the multiple testing threshold are presented as red dots

The results of our EWAS pointed out forty-one probes whose methylation was associated with VEGF-A concentration in cellular extracts (Sup. Table 1). Twenty probes were significant after Holm-Bonferroni adjustment (*P* < 1.6 × 10^−7^). The results for associations between DNA methylation and VEGF-A concentration are shown in Figs. 1 and 2. Manhattan plot shows that methylation is spread across different chromosomes. Chromosome 19 and chromosome 3 showed more significantly associated methylation sites than other chromosomes. The direction of all associations between DNA methylation and VEGF-A is presented with volcano plot.

Table 2 presents the list of twenty CpG sites that were significant after Holm-Bonferroni correction. Location and genes for CpG sites were retrieved from the annotation file of CpGassoc R package (CRAN). Significant *P* values have been detected; however, a small effect size was attributed to each CpG site. Six CpG sites did not have annotated genes. In silico analysis using the Ensembl browser was conducted to localize those CpG sites on the Human Genome (GRCh38.p10), in order to explore their genetic environment and define the nearest genes that could be impacted by CpG methylation. For each of the six CpG sites, the nearest upstream and downstream gene was identified. Results of the in silico analysis are presented in Table 3.

**Table 2:** Novel associations between *VEGFA* levels and DNA methylation in a subset of the STANISLAS Family Study, after Holm-Bonferroni correction (*P* < 1.6 × 10^−07^). Data retrieved from the CpGassoc annotation file

CpG Site	Chromosome	Gene	Location	Mean beta values	Effect size	*P* value
cg05739757	2q11.2	*RPL31*	TSS200	0.07	0.00176	5.64 × 10^−15^
cg23333878	9p24.2	*GLIS3*	5′UTR; 1stExon	0.08	0.00270	3.86 × 10^−14^
cg21838233	20q11.21	*TPX2*	1stExon; 5′UTR	0.04	0.00191	4.01 × 10^−12^
cg18815539	4p15.2	*SEPSECS*	1stExon; 5′UTR	0.04	− 0.00197	1.70 × 10^−12^
cg21968169	12q24.31	*LOC338799*, *SETD1B*	TSS1500	0.09	− 0.00189	1.11 × 10^−12^
cg16333561	7q11.23	ND	ND	0.90	− 0.00130	7.02 × 10^−11^
cg20547575	7q11.22	*AUTS2*	Body	0.03	− 0.00349	1.52 × 10^−10^
cg15014826	19p13.12	ND	ND	0.03	− 0.00157	1.12 × 10^−10^
cg00117600	15q21.3	*PIGB*	5′UTR; 1stExon	0.04	0.00118	8.84 × 10^−09^
cg08759276	16q24.1	ND	ND	0.78	− 0.00153	3.31 × 10^−09^
cg05275012	3p22.1	ND	ND	0.94	0.00176	8.81 × 10^−08^
cg10517202	3q26.32	ND	ND	0.88	− 0.00103	8.74 × 10^−08^
cg09614565	3p14.3	*IL17RD*	TSS200	0.02	− 0.00283	5.69 × 10^−08^
cg13689591	3q21.1–q21.2	*KALRN*	Body	0.88	0.00199	2.58 × 10^−08^
cg06934988	17p13.1	*USP43*	TSS200	0.02	− 0.00188	1.96 × 10^−08^
cg06785213	19q13.4	*HAS1*	TSS200	0.20	0.00087	1.06 × 10^−08^
cg13332754	18q22.3	ND	ND	0.95	0.00164	1.39 × 10^−07^
cg03551607	13q14.2	*ESD*	5′UTR	0.04	0.00115	1.34 × 10^−07^
cg24364967	2p16.1	*CLHC1*	5′UTR	0.12	0.00122	1.22 × 10^−07^
cg15057061	3q26.33	*SOX2OT*	Body	0.04	− 0.00151	1.08 × 10^−07^

*ND* no data, *Mean beta values* mean value of the methylation occurring at the significant CpG sites (1 = methylated, 0 = non methylated), *Effect size* quantitative measure of the magnitude of the methylation effect on VEGF-A concentration

**Table 3:** Supplementary information retrieved by in silico analysis for methylation sites without annotated gene and chromosome position

CpG site (strand)		cg16333561 (−)	cg15014826 (+)	cg08759276 (−)	cg05275012 (−)	cg10517202 (−)	cg13332754 (−)
Chromosome		7p21.2	19p13.12	16q24.1	3p22.1	3q26.32	18q22.3
Nearest genomic feature (product/strand)	Upstream	*AC011287*.*1* (novel lincRNA/+)	*ZSWIM4* (protein coding/+)	*AC009108*.*4* (unknown/+)	*AC122683*.*1* (lincRNA/−)	*LINC00578* (lincRNA/+)	*TSHZ1* (protein coding/+)
	Downstream	*AC005019*.*2* (novel lincRNA/−)	*AC020916*.*1* (lincRNA/−)	*AC009108*.*2* (lincRNA/−)	*HMGN2P24* (pseudogene/−)	*RN7SKP52* (misc RNA/−)	*TSHZ1* (protein coding/+)
Location		7:13803079–13803128	19:13833585–13833634	16:86610656–86610705	3:40619738–40619787	3:177469455–177469504	18:75290150–75290199
Regulatory features (T cells, natural killer cells or B cells)		1 enhancer and 2 CTCF binding sites	8 promoters	4 promoters and 1 CTCF binding site	1 promoter, 1 enhancer and 3 CTCF binding sites	1 promoter; 1 CTCF and 1 enhancer	1 promoter; 2 enhancers and 1 CTCF binding site
Nearest coding gene (strand)	Upstream	*ARL4A* (*+*)	*ZSWIM4* (*+*)	*FOXL1* (*+*)	*ZNF621* (*+*)	*TBL1XR1* (*−*)	*TSHZ1*, *ZADH2* (*+*)
	Downstream	*ETV1* (*−*)	*NANOS3* (*+*)	*C16orf95* (*−*)	*CTNNB1* (*+*)	*KCNMB2* (*+*)	*SMIM21* (*−*)

Furthermore, we have analysed Holm-Bonferroni significant CpG sites using two different principles**.** Firstly, we have studied the genes encoded in the position of the CpG, focusing on their function, relation to diseases and association with VEGF-A (Sup. Table 2). Altogether, there were 28 genes retrieved from the annotation results file of the EWAS analysis and identified from in silico analysis, which were encoded in the proximity of 20 significant CpG sites. None of the genes was directly related to the VEGF-A protein. In order to reveal common genetic pathways, genomic environment of all CpG sites was studied in detail using Ensembl browser. Results are presented in the supplementary data (Sup. Figures 1 and 2). Secondly, we have studied the genetic environment of each CpG schematically, to detect possible common patterns related to the location of CpG on the genome (Sup. Figure 1 and 2).

The overall aim of such analysis was to explore publicly available databases in order to detect common biological pathways between VEGF-A and the concerned genes. Some of the identified genes were previously associated with VEGF-A (Sup. Table 2), but those associations were not explained genetically. DNA methylation could provide an answer to these associations. Furthermore, the patterns in which methylation occurs on the genome could provide us new information on methylation function and pave the way for novel hypotheses that could explain the function of CpGs in non-coding regions or locations without direct relation to a specific phenotype.

**Fig. 3:**
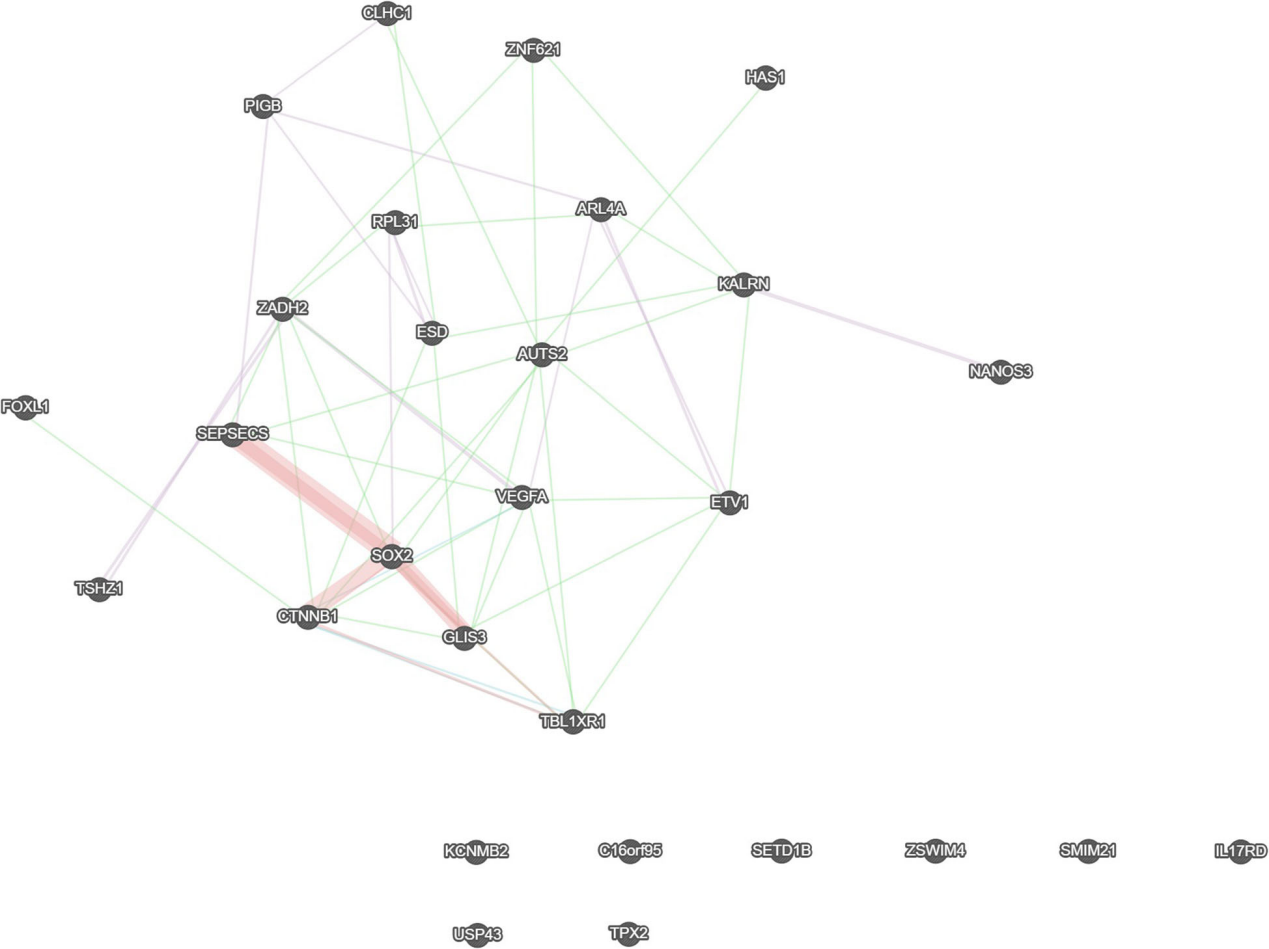
Network of genes related to CpG sites significantly associated with VEGF-A concentrations. Green lines present genetic associations, violet lines present co-expression, blue lines present common pathways and rose lines present physical interactions between connected genes

Interactions between annotated genes were further analysed with GeneMANIA app. GeneMANIA enables the construction of a composite gene-gene functional interaction network from a list of genes collected from many large, publicly available biological datasets [26]. A list of 28 genes has been input into GeneMANIA to research their possible relation to *VEGFA*: *RPL31*, *GLIS3*, *TPX2*, *SEPSECS*, *LOC338799*, *SETD1B*, *AUTS2*, *PIGB*, *IL17RD*, *KALRN*, *USP43*, *HAS1*, *ESD*, *CLHC1*, *SOX2*, *ARL4A*, *ZSWIM4*, *FOXL1*, *ZNF621*, *TBL1XR1*, *TSHZ1*, *ZADH2*, *ETV1*, *NANOS3*, *C16orf95*, *CTNNB1*, *KCNMB2* and *SMIM21*. A gene network created as a result of this is presented in Fig. 3. Some of the input genes were not found by the bioinformatics tool and are thus not presented on the figure. Some genes, i.e. *TPX*, *C16orf95*, *KCNMB2*, *ZSWIM4*, *SETD1B*, *SMIM21*, *IL17RD* and *USP43* were not related to any of genes input into GeneMania and are also not presented on the figure. Results revealed seven genes that were previously observed to have minor interactions with VEGF-A, namely *ARL4A*, *ZADH2*, *SEPSECS*, *CTNNB1*, *TBL1XR1*, *GLIS3* and *ETV1* [27]. *ZADH2*, *SEPSECS*, *CTNNB1*, *TBL1XR1*, *GLIS3* and *ETV1* had minor genetic interactions (presented with a green line in Fig. 3). *ARL4A* and *ZADH2* had similar expression levels with *VEGFA* in gene expression studies (violate lines), as demonstrated in the analysis of the gene expression of the glioblastoma multiforme cancer cells [28] and study of steam cell populations [29], respectively. A blue line between *CTNNB1* and *VEGFA* designates a common pathway, identified in pathway-based analysis of human functional protein network [30]. Most physical protein-protein interactions were detected within *SEPSECS*, *SOX* and *AUTS2* genes (rose lines).

Significant results have been analysed using MethylGSA, a Bioconductor package to identify relevant physiological pathways. The analysis showed that CpG sites identified are involved in numerous molecular processes. The details are presented in the supplementary file (Sup. Figure 3).

## Discussion

We have comprehensively explored the DNA methylome in a population of healthy individuals and have identified 41 methylation sites significantly associated with VEGF-A concentrations derived from PBMC extracts (Sup. Table 1). Significance levels after Bonferroni correction ranged from *P* = 1.08 × 10^−7^ (cg15057061) to *P* = 5.64 × 10^−15^ (cg05739757) for 20 CpG sites. Ten CpGs produced a positive effect size (range, 0.00087 to 0.0027), whereas the remaining ten methylation biomarkers possessed a negative effect size based on VEGF-A concentrations (range, 0.00349 to 0.00103). This study is the first epigenome-wide association study investigating the links between DNA methylation and VEGF-A concentration in a population of healthy individuals and the importance of its findings will be discussed below.

For all Holm-Bonferroni significant CpG sites, we looked for the nearest coding genes to understand the link between methylation of these genes and VEGF-A concentration. Fourteen genes were annotated in the result file obtained after performing EWAS (Table 2) using CpGassoc R package. To find the genomic features within the location of the rest six CpG sites, we have performed in silico analysis using Ensembl browser. None of the CpGs identified in this study was located within or near the *VEGF* gene family or its associated genes (Sup. Table 3). However, some CpGs identified in this study have been previously implicated in VEGF-related biological processes, such as cell proliferation, cell growth, angiogenesis and related diseases (Sup. Table 2). One such relation was found with *TPX2* gene, significantly associated with cg21838233 (*P* = 4.01 × 10^−12^). The *TPX2* gene is overexpressed in colon cancer, leading to vessel invasion and metastasis of colon cancer cells [31]. *TPX2* gene silencing results in the inhibition of cell proliferation, and this effect has been linked to the down-regulation of the *VEGFA* expression [32]. Cg21838233 is located within the promoter region of *TPX2*, where methylation can play a crucial role in the control of gene expression [33]. The degree of methylation and location of the methylation site may directly affect the transcription and subsequent expression of the gene. Therefore, we could hypothesize that the expression of *TPX2* is controlled by methylation of cg21838233, which in turn reflects further in the expression of *VEGFA*. However, such assumptions should be confirmed with other studies.

Another intriguing result was cg06785213 (*P* = 1.06 × 10^−08^) which was found in the vicinity of the *HAS1* gene, 62 bp in 5′ upstream region. The *HAS1* gene family encodes for hyaluronic acid (HA), which has an essential role in tissue development and homeostasis, and directs the initiation and progression of various pathological conditions, including angiogenesis [34]. Both proteins, HAS-1 and VEGF-A, have an important role in antigenic cascade [35, 36]. Thus, methylation in the vicinity of the *HAS1* gene could play a distinct role in regulation of this process. Further research is required to confirm these hypotheses and elucidate new epigenetic pathways.

**Fig. 4:**
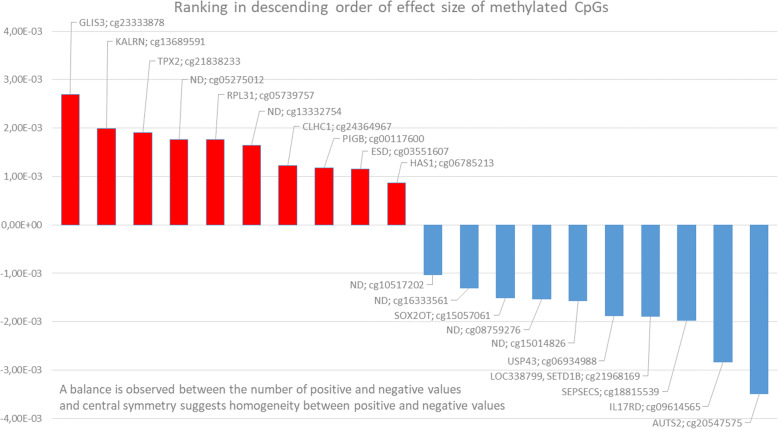
Ranking of the effect sizes of significant CpG sites in descending order

Five other genes in sup. Table 2 were related to angiogenic processes, *namely ARL4A*, *ETV1*, *CTNNB1*, *TBL1XR1* and *TSHZ1*, located in the vicinity of CpGs detected in non-coding regions. In total, 6 out of 20 CpG sites from non-coding regions were significantly associated with VEGF-A (Table 3). Little is known whether such CpGs can have a real impact on genes in their proximity. However, it is known that it is not only the sequence in the immediate proximity of a region, such as promoter, that can influence gene activity [37]. DNA regions that were previously considered as “junk” DNA are now being considered as indispensable elements of regulation of gene expression [37]. Looking upstream and downstream of 6 annotated CpG sites, we have discovered that the most common genetic features in their immediate proximity were long non-coding RNAs (lncRNAs), which are emerging as regulators of gene expression in pathogenesis [38]. Cascade CpG-lincRNAs could take a part in regulation of coding genes (e.g. *ARL4A*, *ETV1* or *CTNNB1*) and could thus impact on VEGF-A regulation. Certainly, all the above assumptions need to be verified. However, it is important that we consider all of the options that might, in the future, elucidate important regulation pathways. The small effect sizes of the significant CpG sites in this study showed that there was no mayor methylation site that would impact on VEGF-A concentration, but there is a sum of the small effect sizes that have a considerable epigenetic effect. Another interesting observation was the repartition of the effect sizes; half of them had positive values while other half had negative values, implying that VEGF-A regulation with methylation works in both directions, towards the increasing or decreasing of VEGF-A concentration (Fig. 4).

Methylation of CpGs, located on gene coding sections of DNA, has the potential to silence gene expression, which is especially important in disease development [39]. Abnormal patterns of DNA methylation have been observed in cancer, most commonly in CpG islands in gene promoter regions [40]. Schematic presentation of genetic regulatory elements in the vicinity of CpGs (Sup. Figure 1 and 2) demonstrated that most CpG sites significantly associated with VEGF-A concentrations were located within promoter regions, a regulatory region of DNA, where transcription is initiated. Normally, CpG islands within promoters are well characterized, but sometimes they are found in deserted areas [41]. However, there is evidence that some orphan CpG islands may initiate transcription and are likely to represent either uncharacterized promoters or promoters driving transcription of non-coding RNA [37]. CTCF binding sites present the second most common element highlighted. They enable CTCF zinc-finger transcription factor to bind and thus activate or repress the activity of various genes; moreover, they can act as enhancer-blocker [42]. Enhancers are the third regulatory elements found in the vicinity of CpGs. It enhances gene transcription by interactions with trans-acting factors, which allows specific control of gene activation, through chromatin looping of the intervening DNA [37].

We have noticed that regulatory elements are becoming less frequent with the distance from a CpG site. It means that identified CpG sites were located within regulatory vivid regions, indicating that CpGs could also be involved as an important element in regulation, without being located directly on the gene coding part.

All of the genes related to the 20 Holm-Bonferroni significant CpGs were also input into GeneMania to explore common genetic and physiological pathways. Seven genes were associated with *VEGFA* (***ARL4A***, *ZADH2*, *SEPSECS*, ***CTNNB1***, ***TBL1XR1***, *GLIS3* and ***ETV1***). For the genes highlighted in bold the relation with VEGF-A has been further confirmed with bibliographical research (Sup. Table 2).

A potential co-localisation of detected CpG sites and VEGF-A related genetic variants was also observed. Though 3 VEGF-A SNPs (i.e. rs10738760 and rs7043199, rs2639990) were localized at the same cytogenetic positions as 2 CpG sites (i.e. cg23333878 and cg13332754, respectively) considerable distances involved show that there is no co-localisation. The comparison of the epigenetic results with our previous GWAS [18, 19] shows that these new results shed additional light on the complexity of the mechanisms involving VEGF-A. The associations highlighted between VEGF-A and the CpG sites, as well as our previous VEGF GWAS [18, 19], all support the high heritability of VEGF-A, both at the genetic and epigenetic level.

## Conclusion

We have found significant associations between DNA methylation and VEGF-A concentrations measured from the PBMCs cellular extracts. Significant CpG sites were located in vicinity of different coding genes, none of which was directly involved in VEGF-A regulation. Replication of these results in independent cohorts is important for their confirmation and could further provide new knowledge that could be used for the development of next-generation medications against VEGF-A-related diseases.

## Methods

### Populations

The SFS is a 10-year longitudinal survey with 3 visits at 5-year intervals, involving 1006 families from Vandoeuvre-lès-Nancy, France, first recruited between 1993 and 1995 [43, 44]. All subjects were of Caucasian origin, without the presence of chronic disorders, e.g. CVD or cancer, or previous personal history of such diseases. The study protocols were approved by the Comité Consultatif pour la Protection des Personnes dans la Recherche Biomédicale de Lorraine (Advisory Committee for the protection of people in biomedical research in Lorraine), and all subjects gave written informed consent for their participation in the study. All experiments were performed in accordance with relevant guidelines and regulations.

### Data collection

Biological and clinical measurements were determined using appropriate, validated procedures. Blood samples were collected between 8 and 9 a.m. after overnight fasting. DNA was extracted by the Miller technique [45] and was stored at − 80 °C until further use. Body mass index (BMI) was calculated as weight (kg) divided by height^2^ (m^2^). All measurements were obtained by trained professionals.

### Biological measurements

#### Isolation of PBMCs

Full blood from healthy donors was collected into sodium heparin tubes. Samples were homogenized with Hanks’ Balanced Salt Solution (SIGMA Aldrich, reference H6648) (*V*_Hanks_ = *V*_blood_) and poured gently into a 15 mL tube with Ficoll^TM^ paque (Sigma Aldrich, reference 17-1440-02) solution (*V*_Ficoll_ = *V*_Hanks_ + *V*_blood_). The contents were centrifuged for 30 min at 300×*g* at room temperature.

High-density PBMC ring was retrieved and collected into a 15-mL tube, filled with Hanks Balanced Salt Solution and centrifuged for 10 min at 1000×*g* at room temperature (first washing). The supernatant was aspirated and 2 mL of Hanks Balanced Salt Solution was added. The tube was filled up to 15 mL with Hanks Balanced Salt Solution and centrifuged for a further 10 min at 1000×*g* at room temperature (second washing). The pellet was collected into an Eppendorf tube with 1 mL of Hanks Balanced Salt Solution. PBMCs populations were evaluated by microscopic observation after May-Grunwald-Giemsa staining and PBMCs concentration was normalized to 10^6^ cells/mL in Hanks Buffer. After final centrifugation of 5 min at 1000×*g* at room temperature, the supernatant was aspirated and the pellet of PBMCs was processed immediately or stored at − 80 °C to maintain stability.

#### Total protein extraction

The lysis solution (lysate) was composed of 320 μL of cell lysis buffer (CelLytic^TM^-M, SIGMA Aldrich, reference C2978) and 1.6 μL of protease inhibitor (0.5%, Protease Inhibitor Cocktail, SIGMA Aldrich, reference P8215) for the samples with counted cells (> 10^6^) and was added to the lymphocyte pellet. The mixture was stirred for 15 min at room temperature and centrifuged for 15 min at 12000×*g* and 4 °C. The supernatant was collected and was immediately used for further analysis or stored at − 80 °C to maintain stability.

#### VEGF-A measurement

PBMC extract concentrations of VEGF-A were estimated using the Randox high-sensitivity multiplex cytokine and growth factor array (Evidence Investigator Analyzer, Randox Laboratories Ltd., Crumlin, UK).

### DNA methylation analysis

#### DNA methylation assay

DNA methylation patterns were investigated using a method, previously described in detail [46, 47]. Briefly, genome-wide methylation profiling of bisulfite-converted genomic DNA was performed by Illumina HumanMethylation450 bead array (Illumina Inc., San Diego, CA, USA). Illumina is using Infinium I and II arrays with probes for detection of methylated and unmethylated CpG sites. Methylation ratio, referred to as beta value by Ilumina’s software, is the proportion methylated/(methylated + unmethylated) for each CpG in the population of cells from which we extracted DNA.

#### Quality control

R package minfi (Bioconductor) was used to analyse and visualize Illumina Infinium methylation arrays [48]. The first step in microarray data preprocessing consisted of removing all probes that can generate artifactual data. Firstly, a detection *P* value was assigned to each probe. High detection *P* value normally corresponds to a probe with a low quality signal; therefore, probes with *P* > 0.05 were removed from all samples. Furthermore, probes missing in > 5% of the samples were excluded. To avoid spurious associations, probes containing locations on the genome where variation is already annotated in HumanMethlyation450 annotation file IlluminaHumanMethylation450kanno.ilmn12.hg19 (i.e. probes containing single-nucleotide polymorphism (SNP), sex chromosomes and a single-base extension (SBE) site) were excluded. Finally, probes containing cross-reactive and target polymorphic CpGs [49] were excluded, leaving 314 440 probes out of 484 777 for statistical analysis. In addition, one individual was excluded from our cohort after quality control checks of methylation array data (outlier of plotted median of the methylated against unmethylated intensity), leaving 200 individuals for the analysis.

#### Normalization

Second step in microarray data preprocessing was removing sources of variation, related to technical limitations—data normalization. Background correction, colour bias (dye bias) adjustment and Infinium I/II bias correction were carried out with Illumina background correction and SWAN [50] in the R package minfi.

#### Association study

CpGassoc (CRAN) was used to test for association between methylation at CpG sites across the genome and VEGF-A concentration in PBMC extracts [51]. As VEGF-A concentrations were not normally distributed in our population, a log-transformation has been applied to normalize the distribution. The random mixed-effects model included gender, age, BMI, family structure and individual blood cell counts (neutrophils, lymphocytes, monocytes, eosinophils and basophils) as covariates and chip array as random effect. In our model, cell counts were added as additional covariate terms to control for the confounding effects of variable leukocyte distribution for examination of the association between DNA methylation and VEGF-A concentration. Holm-Bonferroni correction for multiple testing was applied to the result.

### In silico analysis

Ensembl browser [52] was used for localization of CpG sites on the Human Genome (GRCh38.p10), as well as for the establishment of regulatory features from their genomic environment. All annotated genes were investigated for interactions with VEGF-A gene using cytoscape app GeneMANIA [26]. MethylGSA R-package was used to relate significant genes or CpGs to known biological properties [53].

## Supplementary information

**Additional file 1: Sup. Figure 1.** Genomic environment of six CpG sites. Dark green regions present a CpG site, numbers on left and right of the box indicate a location, within which CpG can be found. Nearest genomic features upstream (left) or downstream (right) are presented for each CpG. Distance (bp) between each CpG and genomic feature is indicated in light green regions. Turquoise squares present CTCF regions, red promoter region and yellow enhancers. Square brackets [ ] indicate that CpG is located within genomic feature. Seven different PBMC cell types were looked up (Sup. Table 1). Number 1-7/7 in each box of particular genomic region is indicating in what extent this genomic feature is presented in PBMCs. Diagrams on the top are presenting patterns of genomic features that can be found in the genomic environment of CpGs. We can see that in the immediate proximity of CpG enhancers are the most common and that with distance, genetic features become less common (regions of non-coding DNA). **Sup. Figure 2.** Genetic environment of fourteen CpG sites. **Sup. Table 1.** Forty-one significant CpG sites related to VEGF concentration derived from PBMCs extracts. **Sup. Table 2.** Summary table explaining the potential functionality and biological plausibility of each of the 20 significant CpGs and their nearby genes. **Sup. Table 3.** List of VEGF genes, VEGF receptor genes and VEGF-A-related genes. Genes in direct relation to VEGF-A were determined with STRING tool (http://version10.string-db.org/), the location was retrieved using Ensembl (www.ensembl.org/). **Sup. Figure 3.** Analysis of significant CpG sites. MethylGSA, a Bioconductor package was used to find relevant physiological pathways. Significant results are presented in the figure.

## Data Availability

The datasets used and/or analysed during the current study are available from the corresponding author on reasonable request.

## Abbreviations

BMI: Body mass index
COPD: Chronic obstructive pulmonary disease
DNA: Deoxyribonucleic acid
EWAS: Epigenome-wide association studies
GWAS: Genome-wide association studies
PBMC: Peripheral blood mononuclear cell
SBE: Single-base extension
SD: Standard deviation
SFS: STANISLAS Family Study
SNP: Single-nucleotide polymorphism
TSS: Transcription start site
VEGF: Vascular endothelial growth factor
