# Supplementary material for: Epigenome-wide association study in healthy individuals identifies significant associations with DNA methylation and PBMC extract VEGF-A concentration
Source: Clin Epigenetics. 2020 Jun 5;12:79. doi: 10.1186/s13148-020-00874-w (PMC7273671; doi:10.1186/s13148-020-00874-w)
Supplement: Supplementary file 1 — Additional file 1: Sup. Figure 1. Genomic environment of six CpG sites. Dark green regions present a CpG site, numbers on left and right of the box indicate a location, within which CpG can be found. Nearest genomic features upstream (left) or downstream (right) are presented for each CpG. Distance (bp) between each CpG and genomic feature is indicated in light green regions. Turquoise squares present CTCF regions, red promoter region and yellow enhancers. Square brackets [ ] indicate that CpG is located within genomic feature. Seven different PBMC cell types were looked up (Sup. Table 1). Number 1-7/7 in each box of particular genomic region is indicating in what extent this genomic feature is presented in PBMCs. Diagrams on the top are presenting patterns of genomic features that can be found in the genomic environment of CpGs. We can see that in the immediate proximity of CpG enhancers are the most common and that with distance, genetic features become less common (regions of non-coding DNA). Sup. Figure 2. Genetic environment of fourteen CpG sites. Sup. Table 1. Forty-one significant CpG sites related to VEGF concentration derived from PBMCs extracts. Sup. Table 2. Summary table explaining the potential functionality and biological plausibility of each of the 20 significant CpGs and their nearby genes. Sup. Table 3. List of VEGF genes, VEGF receptor genes and VEGF-A-related genes. Genes in direct relation to VEGF-A were determined with STRING tool (http://version10.string-db.org/), the location was retrieved using Ensembl (www.ensembl.org/). Sup. Figure 3. Analysis of significant CpG sites. MethylGSA, a Bioconductor package was used to find relevant physiological pathways. Significant results are presented in the figure. [file 13148_2020_874_MOESM1_ESM.docx]

**SUPPLEMENTARY DATA**

***Epigenome-wide association study in healthy individuals identifies significant associations with DNA methylation and PBMCs extracts VEGF-A concentration***

Vesna Gorenjak^1^, Dwaine R. Vance^2^, Sébastien Dade^1^, Maria G. Stathopoulou^1^, Lauren Doherty^2^, Ting Xie^1^, Helena Murray^2^, Christine Masson^1^, John Lamont^2^, Peter Fitzgerald^2^, Sophie Visvikis-Siest^¥, 1, 3^

^1^ Université de Lorraine, Inserm, IGE-PCV, F-54000 Nancy, France

^2^ Randox Laboratories Limited, Crumlin, Co. Antrim, Northern Ireland, United Kingdom

^3^ Department of Internal Medicine and Geriatrics, CHU Technopôle Nancy-Brabois, Rue du Morvan, F-54511, Vandoeuvre-lès-Nancy, France

**^¥^Corresponding author:**

Dr. Sophie VISVIKIS-SIEST

INSERM UMR U1122; IGE-PCV, Faculté de Pharmacie – Université de Lorraine

30 rue Lionnois

54000 Nancy, France

Tel: +33.6.07.60.25.69

E-mail address: [sophie.visvikis-siest@inserm.fr](mailto:sophie.visvikis-siest@inserm.fr)

**Sup. Figure 1:** **Genomic environment of six CpG sites.** *Dark green regions present a CpG site, numbers on left and right of the box indicate a location, within which CpG can be found. Nearest genomic features upstream (left) or downstream (right) are presented for each CpG. Distance (bp) between each CpG and genomic feature is indicated in light green regions. Turquoise squares present CTCF regions, red promoter region and yellow enhancers. Square brackets [ ] indicate that CpG is located within genomic feature. Seven different PBMC cell types were looked up (Sup. Table 1). Number 1-7/7 in each box of particular genomic region is indicating in what extent this genomic feature is presented in PBMCs. Diagrams on the top are presenting patterns of genomic features that can be found in the genomic environment of CpGs. We can see that in the immediate proximity of CpG enhancers are the most common and that with distance, genetic features become less common (regions of non-coding DNA).*

**Sup. Figure 2:** *Genetic environment of fourteen CpG sites.*

**Sup. Table 1:** *Forty-one significant CpG sites related to VEGF concentration derived from PBMCs extracts.*

| CpG Labels | P-value | Holm. Sig. | FDR | Effect size | Chromosome | Strand |
| --- | --- | --- | --- | --- | --- | --- |
| cg15144153 | 2.80 x 10^ -07 | FALSE | 0.00400 | 0.00200 | chr2 | - |
| cg24364967 | 1.22 x 10^ -07 | TRUE | 0.00214 | 0.00122 | chr2 | - |
| cg15057061 | 1.08 x 10^ -07 | TRUE | 0.00200 | -0.00151 | chr3 | - |
| cg23012579 | 4.29 x 10^ -07 | FALSE | 0.00519 | -0.00178 | chr5 | - |
| cg18262852 | 1.25 x 10^ -06 | FALSE | 0.01312 | 0.00081 | chr11 | + |
| cg03551607 | 1.34 x 10^ -07 | TRUE | 0.00219 | 0.00115 | chr13 | + |
| cg06934988 | 1.96 x 10^ -08 | TRUE | 0.00051 | -0.00188 | chr17 | - |
| cg17949256 | 3.20 x 10^ -07 | FALSE | 0.00422 | -0.00123 | chr17 | + |
| cg03521258 | 2.87 x 10^ -06 | FALSE | 0.02492 | -0.00183 | chr19 | + |
| cg05739757 | 5.64 x 10^ -15 | TRUE | 1.77 x 10^ -09 | 0.00177 | chr2 | - |
| cg05275012 | 8.81 x 10^ -08 | TRUE | 0.00173 | 0.00176 | chr3 | + |
| cg09614565 | 5.69 x 10^ -08 | TRUE | 0.00128 | -0.00283 | chr3 | + |
| cg10517202 | 8.74 x 10^ -08 | TRUE | 0.00173 | -0.00104 | chr3 | + |
| cg13689591 | 2.58 x 10^ -08 | TRUE | 0.00062 | 0.00200 | chr3 | + |
| cg18815539 | 1.70 x 10^ -12 | TRUE | 1.347 x 10^ -07 | -0.00197 | chr4 | + |
| cg03663456 | 6.32 x 10^ -07 | FALSE | 0.00709 | 0.00144 | chr7 | - |
| cg16333561 | 7.02 x 10^ -11 | TRUE | 3.68 x 10^ -06 | -0.00130 | chr7 | - |
| cg20547575 | 1.52 x 10^ -10 | TRUE | 5.96 x 10^ -06 | -0.00350 | chr7 | - |
| cg12141623 | 1.83 x 10^ -06 | FALSE | 0.01658 | -0.00116 | chr8 | - |
| cg19497501 | 6.06 x 10^ -06 | FALSE | 0.04651 | 0.00068 | chr8 | - |
| cg20085411 | 1.84 x 10^ -06 | FALSE | 0.01658 | 0.00099 | chr8 | + |
| cg22735324 | 2.76 x 10^ -07 | FALSE | 0.00400 | 0.00155 | chr8 | - |
| cg13496481 | 6.02 x 10^ -06 | FALSE | 0.04651 | 0.00321 | chr9 | - |
| cg23333878 | 3.86 x 10^ -14 | TRUE | 6.06 x 10^ -09 | 0.00270 | chr9 | - |
| cg04843252 | 4.20 x 10^ -07 | FALSE | 0.00519 | 0.00104 | chr10 | - |
| cg10928925 | 1.33 x 10^ -06 | FALSE | 0.01348 | -0.00091 | chr10 | + |
| cg19004007 | 1.25 x 10^ -06 | FALSE | 0.01312 | 0.00134 | chr10 | - |
| cg19864972 | 1.46 x 10^ -06 | FALSE | 0.01438 | 0.00126 | chr10 | + |
| cg14682277 | 3.09 x 10^ -06 | FALSE | 0.02555 | -0.00099 | chr11 | - |
| cg21968169 | 1.11 x 10^ -12 | TRUE | 1.16 x 10^ -07 | -0.00189 | chr12 | - |
| cg18073151 | 2.93x 10^ -06 | FALSE | 0.02492 | -0.00220 | chr14 | - |
| cg00117600 | 8.84 x 10^ -09 | TRUE | 0.00028 | 0.00118 | chr15 | - |
| cg03790192 | 3.22 x 10^ -07 | FALSE | 0.00422 | -0.00131 | chr16 | + |
| cg08759276 | 3.31 x 10^ -09 | TRUE | 0.00012 | -0.00153 | chr16 | + |
| cg16698748 | 1.67 x 10^ -06 | FALSE | 0.01593 | 0.00072 | chr16 | - |
| cg13332754 | 1.39 x 10^ -07 | TRUE | 0.00219 | 0.00164 | chr18 | + |
| cg06785213 | 1.06 x 10^ -08 | TRUE | 0.00030 | 0.00087 | chr19 | - |
| cg15014826 | 1.12 x 10^ -10 | TRUE | 5.04 x 10^ -06 | -0.00157 | chr19 | - |
| cg25492620 | 5.26 x 10^ -07 | FALSE | 0.00612 | -0.00154 | chr19 | - |
| cg21838233 | 4.01 x 10^ -12 | TRUE | 2.52 x 10^ -07 | 0.00191 | chr20 | - |
| cg06295071 | 5.99 x 10^ -06 | FALSE | 0.04651 | 0.00241 | chr21 | - |

**Sup. Table 2:** *Summary table explaining the potential functionality and biological plausibility of each of the 20 significant CpGs and their nearby genes.*

|  | Nearest Coding Gene(s) | Function | Disease | Interactions with VEGF |
| --- | --- | --- | --- | --- |
| cg05739757 | RPL31 | Encodes a ribosomal protein | Increased expression in colorectal cancer (1) and in prostate cancer (2) | None identified |
| cg23333878 | GLIS3 | Acts as both a repressor and activator of transcription | Neonatal diabetes mellitus, with congenital hypothyroidism (3) | None identified |
| cg21838233 | TPX2 | Required for spindle fibre development | Overexpressed in gastric cancer cells (4) | TPX2 gene silencing inhibit proliferation and invasion of colon cancer cells, possible related to down-regulation of VEGF gene (5) |
| cg18815539 | SEPSECS | Implicated in the catalysation of O-phosphoseryl-tRNA (Sec) to selenocysteinyl-tRNA (Sec) | Pontocerebellar hypoplasia type 2D (6) | None identified |
| cg21968169 | LOC338799  SETD1B | Long intergenic non-protein coding RNA 1089  A component of a histone methyltransferase complex that produces trimethylated H3 at Lys4 | Hormone‐independent breast cancer,serves as a driver of mammary cell migration (7)  Gastric and colorectal cancers (8) | None identified  None identified |
| cg16333561  (Located in enhancer region) | ARL4A  ETV1 | A member of the ADP-ribosylation factor family of GTP-binding proteins  ETS proteins regulate many target genes that modulate biological processes like cell growth, angiogenesis, migration, proliferation and differentiation. | None identified  Overexpressed in prostate cancer, melanoma and gastrointestinal stromal tumor (9) | ARL4A has been differentially regulated in LV165 and ML20 in response to bevacizumab treatment (10)  EWS-ETS (ETS-family of ETV1 protein) fusion proteins upregulate the transcription of VEGF, overexpressed in Ewing tumors (11) |
| cg20547575 | AUTS2 | Activator of transcription and development regulator | Neurological disorders, autism, overexpressed in liver metastases of pancreatic cancer (12) | None identified |
| cg15014826  (Located in enhancer region) | ZSWIM4  NANOS3 | Protein coding gene for zinc finger SWIM-Type Containing 4  Protein coding gene for Nanos C2HC-Type zinc finger 3, maintenance of the undifferentiated state of germ cells, affects cell proliferation | None identified  Ovarian failure (13), male sterility (14), lung tumor (15) | None identified  None identified |
| cg00117600 | PIGB | Required for the development of a glycosyl-phosphatidylinositol anchor, a glycolipid serving to anchor proteins to the cell surface | **Hyperphosphatasia mental retardation syndrome (16)** | None identified |
| cg08759276  (Located in enhancer region) | FOXL1  C16orf95 | Play critical roles in the regulation of metabolism, cell proliferation and gene expression during ontogenesis  Protein coding gene for chromosome 16 open reading frame 95 | Overexpression of FOXL1 has protective role in breast (17), gastric (18), bladder (19), renal (20) cancer  Bilateral vesicoureteral Reflux (21) | None identified  None identified |
| cg05275012 | ZNF621  CTNNB1 | Zinc finger protein is involved in transcriptional regulation  Part of a complex of proteins that constitute adherens junctions, regulators of epithelia cell growth and adhesion between cells | None identified  Colorectal cancer, pilomatrixoma, medulloblastoma, and ovarian cancer (22) | None identified  VEGF-A expression is regulated by beta-catenin (CTNNB1) in colon cancer (23, 24); VEGF-D mRNA is down-modulated by beta-catenin (25) |
| cg10517202  (Located in enhancer region) | TBL1XR1  KCNMB2 | Required for transcriptional activation by a variety of transcription factors mediated by nuclear receptors  Involved in the control of smooth muscle tone and neuronal excitability | Ovarian cancer (26), autism Spectrum Disorder, Intellectual disability, tumours, Pierpont syndrome (27)  rs7620503 associated with central corneal thickness | Silencing of TBL1XR1 decreased VEGF-C expression - TBL1XR1 may function as an upstream regulator of VEGF-C (26)  None identified |
| cg09614565 | IL17RD | Encodes a component of the interleukin 17 receptor signalling complex | Hypogonadotropic Hypogonadism (28) | None identified |
| cg13689591  (Located in enhancer region) | KALRN | This gene is involved in the trafficking of vesicles. Its protein, Kalirin has been shown to be atheroprotective | Early onset coronary heart disease (29), Huntington’s disease | None identified |
| cg06934988 | USP43 | Involved in the processing of poly-ubiquitin precursors as well as that of ubiquitinated proteins | None identified | None identified |
| cg06785213 | HAS1 | Encodes predicted plasma membrane proteins with multiple transmembrane domains | Inflammatory and degenerative arthropathies such as rheumatoid arthritis, esophageal squamous cell carcinoma | HAS1-siRNA restrains the expression of VEGF (30) |
| cg13332754 | TSHZ1  SMIM21 | 2H2-type zinc-finger protein family, involved in transcriptional regulation of developmental processes  Protein coding gene for small integral membrane protein | Congenital aural atresia syndrome (31)  None | Circulating VEG levels in GWAS were related to variant in chromosome 18, located in an intergenic region downstream of the ZADH2 gene and upstream of the Teashirt Zinc Finger Homeobox 1 (TSHZ1) gene (32)  None |
| cg03551607 | ESD | Enzyme involved in the recycling of sialic acids | Retinoblastoma (33) and Wilson's disease | None |
| cg24364967 | C2orf63  (CLHC1) | Unknown | None | None |
| cg15057061 | SOX2OT | Transcription factor involved in embryonic development and cell fate determination | Optic nerve hypoplasia, breast cancer (34) | None |

Information gathered in the supplementary Table 3 were retrieved from public databases such as NCBI (<https://www.ncbi.nlm.nih.gov/gene/>), WikiGenes (<https://www.wikigenes.org/>) and GeneCards (<http://www.genecards.org/>).

**Sup. Table 3:** *List of VEGF genes, VEGF receptor genes and VEGF-A-related genes. Genes in direct relation to VEGF-A were determined with STRING tool (*[*http://version10.string-db.org/*](http://version10.string-db.org/)*), the location was retrieved using Ensembl (*[*www.ensembl.org/*](http://www.ensembl.org/)*).*

| Gene Name | Chromosome | Cytogenic Band | Location |
| --- | --- | --- | --- |
| *VEGF-A* | 6 | p21.1 | 43,770,183-43,786,487 (+) |
| *VEGF-B* | 11 | q13.1 | 64,234,538-64,238,793 (+) |
| *VEGF-C* | 4 | q34.3 | 176,683,538-176,792,727 (-) |
| *VEGF-D* | X | p22.2 | 15,345,591-15,384,376 (-) |
| *PGF* | 14 | q24.3 | 74,941,834-74,955,784 (-) |
| *FLT1 (VEGFR-1)* | 13 | q12.3 | 28,300,344-28,495,145 (-) |
| *KDR (VEGFR-2)* | 4 | q12 | 55,078,477-55,125,589 (-) |
| *FLT4 (VEGFR-3)* | 5 | q35.3 | 180,601,506-180,650,271 (-) |
| *NRP1* | 10 | p11.22 | 33,177,492-33,336,262 (-) |
| *HIF1A* | 14 | q23.2 | 61,695,401-61,748,259 (+) |
| *VWF* | 12 | p13.31 | 12:5,948,874-6,124,770 (-) |
| *TGFB1* | 19 | q13.2 | 41,301,587-41,353,933 (-) |
| *SRC* | 20 | q11.23 | 37,344,685-37,406,050 (+) |
| *IGF1* | 12 | q23.2 | 102,395,867-102,481,786 (-) |
| *MMP9* | 20 | q13.12 | 46,008,908-46,016,561 (+) |

Methylation of the *VEGFA* gene has been observed in previous studies (30, 31). Moreover, research has shown that VEGF-A receptors KDR and FLT4 are also silenced by DNA methylation. However, if the promoters are unmethylated, other factors (*e.g.* transactivation factors) determine the extent of KDR and FLT4 expression (24, 32).

**Sup. Figure 3: Analysis of significant CpG sites.** *MethylGSA, a Bioconductor package was used to find relevant physiological pathways. Significant results are presented in the figure.*


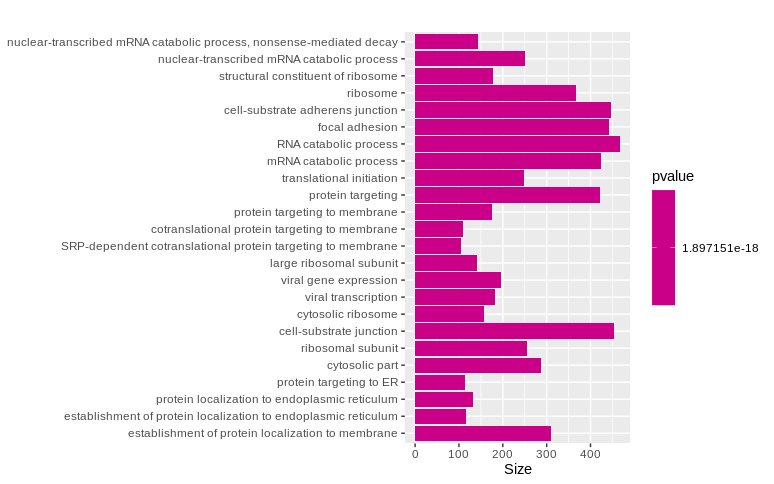


**REFERENCES**

1. K. A. Chester, L. Robson, R. H. Begent, I. C. Talbot, J. H. Pringle, L. Primrose, A. J. Macpherson, G. Boxer, P. Southall and A. D. Malcolm: Identification of a human ribosomal protein mRNA with increased expression in colorectal tumours. *Biochim Biophys Acta*, 1009(3), 297-300 (1989)

2. Y. Maruyama, T. Miyazaki, K. Ikeda, T. Okumura, W. Sato, K. Horie-Inoue, K. Okamoto, S. Takeda and S. Inoue: Short hairpin RNA library-based functional screening identified ribosomal protein L31 that modulates prostate cancer cell growth via p53 pathway. *PLoS One*, 9(10), e108743 (2014) doi:10.1371/journal.pone.0108743

3. V. Senee, C. Chelala, S. Duchatelet, D. Feng, H. Blanc, J. C. Cossec, C. Charon, M. Nicolino, P. Boileau, D. R. Cavener, P. Bougneres, D. Taha and C. Julier: Mutations in GLIS3 are responsible for a rare syndrome with neonatal diabetes mellitus and congenital hypothyroidism. *Nat Genet*, 38(6), 682-7 (2006) doi:10.1038/ng1802

4. C. Tomii, M. Inokuchi, Y. Takagi, T. Ishikawa, S. Otsuki, H. Uetake, K. Kojima and T. Kawano: TPX2 expression is associated with poor survival in gastric cancer. *World J Surg Oncol*, 15 (2017) doi:10.1186/s12957-016-1095-y

5. J. JIAN, Y. HUANG, L.-z. LIU, S. LI and F. DENG: TPX2 gene-silencing inhibits the proliferation and invasion of human colon cancer SW480 cells. *TUMOR*, 36(6), 628-634 (2016)

6. K. Iwama, M. Sasaki, S. Hirabayashi, C. Ohba, E. Iwabuchi, S. Miyatake, M. Nakashima, N. Miyake, S. Ito, H. Saitsu and N. Matsumoto: Milder progressive cerebellar atrophy caused by biallelic SEPSECS mutations. *J Hum Genet*, 61(6), 527-31 (2016) doi:10.1038/jhg.2016.9

7. A. Sas‐Chen, M. R. Aure, L. Leibovich, S. Carvalho, Y. Enuka, C. Körner, M. Polycarpou‐Schwarz, S. Lavi, N. Nevo, Y. Kuznetsov, J. Yuan, F. Azuaje, I. Ulitsky, S. Diederichs, S. Wiemann, Z. Yakhini, V. N. Kristensen, L. Børresen‐Dale A‐, Y. Yarden, T. Sauer, J. Geisler, S. Hofvind, T. F. Bathen, E. Borgen, O. Engebråten, Ø. Fodstad, Ø. Garred, G. A. Geitvik, R. Kåresen, B. Naume, G. M. Mælandsmo, H. G. Russnes, E. Schlichting, T. Sørlie, O. C. Lingjærde, K. K. Sahlberg, H. K. Skjerven and B. Fritzman. *EMBO Mol Med*, 8(9), 1052-64 (2016) doi:10.15252/emmm.201606198

8. Y. J. Choi, H. R. Oh, M. R. Choi, M. Gwak, C. H. An, Y. J. Chung, N. J. Yoo and S. H. Lee: Frameshift mutation of a histone methylation-related gene SETD1B and its regional heterogeneity in gastric and colorectal cancers with high microsatellite instability. *Hum Pathol*, 45(8), 1674-81 (2014) doi:10.1016/j.humpath.2014.04.013

9. B. G. Jang, H. E. Lee and W. H. Kim: ETV1 mRNA is specifically expressed in gastrointestinal stromal tumors. *Virchows Arch*, 467(4), 393-403 (2015) doi:10.1007/s00428-015-1813-9

10. Y. Gokmen-Polar, C. P. Goswami, R. A. Toroni, K. L. Sanders, R. Mehta, U. Sirimalle, B. Tanasa, C. Shen, L. Li, M. Ivan, S. Badve and G. W. Sledge, Jr.: Gene Expression Analysis Reveals Distinct Pathways of Resistance to Bevacizumab in Xenograft Models of Human ER-Positive Breast Cancer. *J Cancer*, 5(8), 633-45 (2014) doi:10.7150/jca.8466

11. B. Fuchs, C. Y. Inwards and R. Janknecht: Vascular endothelial growth factor expression is up-regulated by EWS-ETS oncoproteins and Sp1 and may represent an independent predictor of survival in Ewing's sarcoma. *Clin Cancer Res*, 10(4), 1344-53 (2004)

12. Y. Han, G. Q. Ru, X. Mou, H. J. Wang, Y. Ma, X. L. He, Z. Yan and D. Huang: AUTS2 is a potential therapeutic target for pancreatic cancer patients with liver metastases. *Med Hypotheses*, 85(2), 203-6 (2015) doi:10.1016/j.mehy.2015.04.029

13. M. G. Santos, A. Z. Machado, C. N. Martins, S. Domenice, E. M. Costa, M. Y. Nishi, B. Ferraz-de-Souza, S. A. Jorge, C. A. Pereira, F. C. Soardi, M. P. de Mello, A. T. Maciel-Guerra, G. Guerra-Junior and B. B. Mendonca: Homozygous inactivating mutation in NANOS3 in two sisters with primary ovarian insufficiency. *Biomed Res Int*, 2014, 787465 (2014) doi:10.1155/2014/787465

14. K. Kusz, L. Tomczyk, A. Spik, A. Latos-Bielenska, P. Jedrzejczak, L. Pawelczyk and J. Jaruzelska: NANOS3 gene mutations in men with isolated sterility phenotype. *Mol Reprod Dev*, 76(9), 804 (2009) doi:10.1002/mrd.21070

15. S. Grelet, V. Andries, M. Polette, C. Gilles, K. Staes, A. P. Martin, C. Kileztky, C. Terryn, V. Dalstein, C. W. Cheng, C. Y. Shen, P. Birembaut, F. Van Roy and B. Nawrocki-Raby: The human NANOS3 gene contributes to lung tumour invasion by inducing epithelial-mesenchymal transition. *J Pathol*, 237(1), 25-37 (2015) doi:10.1002/path.4549

16. Y. Murakami, N. Kanzawa, K. Saito, P. M. Krawitz, S. Mundlos, P. N. Robinson, A. Karadimitris, Y. Maeda and T. Kinoshita: Mechanism for release of alkaline phosphatase caused by glycosylphosphatidylinositol deficiency in patients with hyperphosphatasia mental retardation syndrome. *J Biol Chem*, 287(9), 6318-25 (2012) doi:10.1074/jbc.M111.331090

17. J. Zhong, H. Wang, J. Yu, J. Zhang and H. Wang: Overexpression of Forkhead Box L1 (FOXL1) Inhibits the Proliferation and Invasion of Breast Cancer Cells. *Oncol Res*, 25(6), 959-965 (2017) doi:10.3727/096504016x14803482769179

18. Z. Ertao, C. Jianhui, C. Chuangqi, Q. Changjiang, C. Sile, H. Yulong, C. Shirong and W. Hui: Low level of FOXL1 indicates a worse prognosis for gastric cancer patients. *Tumour Biol*, 37(8), 11331-7 (2016) doi:10.1007/s13277-016-4890-8

19. Y. Qin, W. Gong, M. Zhang, J. Wang, Z. Tang and Z. Quan: Forkhead box L1 is frequently downregulated in gallbladder cancer and inhibits cell growth through apoptosis induction by mitochondrial dysfunction. *PLoS One*, 9(7), e102084 (2014) doi:10.1371/journal.pone.0102084

20. F. Q. Yang, F. P. Yang, W. Li, M. Liu, G. C. Wang, J. P. Che, J. H. Huang and J. H. Zheng: Foxl1 inhibits tumor invasion and predicts outcome in human renal cancer. *Int J Clin Exp Pathol*, 7(1), 110-22 (2014)

21. M. G. Butler, S. L. Dagenais, J. L. Garcia-Perez, P. Brouillard, M. Vikkula, P. Strouse, J. W. Innis and T. W. Glover: Microcephaly, intellectual impairment, bilateral vesicoureteral reflux, distichiasis, and glomuvenous malformations associated with a 16q24.3 contiguous gene deletion and a Glomulin mutation. *Am J Med Genet A*, 158a(4), 839-49 (2012) doi:10.1002/ajmg.a.35229

22. K. Na, E. K. Kim, W. Jang and H. S. Kim: CTNNB1 Mutations in Ovarian Microcystic Stromal Tumors: Identification of a Novel Deletion Mutation and the Use of Pyrosequencing to Identify Reported Point Mutation. *Anticancer Res*, 37(6), 3249-3258 (2017) doi:10.21873/anticanres.11688

23. V. Easwaran, S. H. Lee, L. Inge, L. Guo, C. Goldbeck, E. Garrett, M. Wiesmann, P. D. Garcia, J. H. Fuller, V. Chan, F. Randazzo, R. Gundel, R. S. Warren, J. Escobedo, S. L. Aukerman, R. N. Taylor and W. J. Fantl: beta-Catenin regulates vascular endothelial growth factor expression in colon cancer. *Cancer Res*, 63(12), 3145-53 (2003)

24. M. Grazia Lampugnani, A. Zanetti, M. Corada, T. Takahashi, G. Balconi, F. Breviario, F. Orsenigo, A. Cattelino, R. Kemler, T. O. Daniel and E. Dejana: Contact inhibition of VEGF-induced proliferation requires vascular endothelial cadherin, beta-catenin, and the phosphatase DEP-1/CD148. *J Cell Biol*, 161(4), 793-804 (2003) doi:10.1083/jcb.200209019

25. M. Orlandini, S. Semboloni and S. Oliviero: Beta-catenin inversely regulates vascular endothelial growth factor-D mRNA stability. *J Biol Chem*, 278(45), 44650-6 (2003) doi:10.1074/jbc.M304255200

26. M. Ma and N. Yu: Over-Expression of TBL1XR1 Indicates Poor Prognosis of Serous Epithelial Ovarian Cancer. *Tohoku J Exp Med*, 241(3), 239-247 (2017) doi:10.1620/tjem.241.239

27. A. Slavotinek, H. Pua, U. Hodoglugil, J. Abadie, J. Shieh, J. Van Ziffle, M. Kvale, H. Lee, P. Y. Kwok, N. Risch and M. Sabbadini: Pierpont syndrome associated with the p.Tyr446Cys missense mutation in TBL1XR1. *Eur J Med Genet*, 60(10), 504-508 (2017) doi:10.1016/j.ejmg.2017.07.003

28. H. Miraoui, A. A. Dwyer, G. P. Sykiotis, L. Plummer, W. Chung, B. Feng, A. Beenken, J. Clarke, T. H. Pers, P. Dworzynski, K. Keefe, M. Niedziela, T. Raivio, W. F. Crowley, Jr., S. B. Seminara, R. Quinton, V. A. Hughes, P. Kumanov, J. Young, M. A. Yialamas, J. E. Hall, G. Van Vliet, J. P. Chanoine, J. Rubenstein, M. Mohammadi, P. S. Tsai, Y. Sidis, K. Lage and N. Pitteloud: Mutations in FGF17, IL17RD, DUSP6, SPRY4, and FLRT3 are identified in individuals with congenital hypogonadotropic hypogonadism. *Am J Hum Genet*, 92(5), 725-43 (2013) doi:10.1016/j.ajhg.2013.04.008

29. L. Wang, E. R. Hauser, S. H. Shah, M. A. Pericak-Vance, C. Haynes, D. Crosslin, M. Harris, S. Nelson, A. B. Hale, C. B. Granger, J. L. Haines, C. J. Jones, D. Crossman, D. Seo, S. G. Gregory, W. E. Kraus, P. J. Goldschmidt-Clermont and J. M. Vance: Peakwide mapping on chromosome 3q13 identifies the kalirin gene as a novel candidate gene for coronary artery disease. *Am J Hum Genet*, 80(4), 650-63 (2007) doi:10.1086/512981

30. G. Wang, W. Zhao, X. Gao, D. Zhang, Y. Li, Y. Zhang and W. Li: HNF1AAS1 promotes growth and metastasis of esophageal squamous cell carcinoma by sponging miR214 to upregulate the expression of SOX-4. *Int J Oncol*, 51(2), 657-667 (2017) doi:10.3892/ijo.2017.4034

31. I. Feenstra, L. E. Vissers, R. J. Pennings, W. Nillessen, R. Pfundt, H. P. Kunst, R. J. Admiraal, J. A. Veltman, C. M. van Ravenswaaij-Arts, H. G. Brunner and C. W. Cremers: Disruption of teashirt zinc finger homeobox 1 is associated with congenital aural atresia in humans. *Am J Hum Genet*, 89(6), 813-9 (2011) doi:10.1016/j.ajhg.2011.11.008

32. S. H. Choi, D. Ruggiero, R. Sorice, C. Song, T. Nutile, A. Vernon Smith, M. P. Concas, M. Traglia, C. Barbieri, N. C. Ndiaye, M. G. Stathopoulou, V. Lagou, G. B. Maestrale, C. Sala, S. Debette, P. Kovacs, L. Lind, J. Lamont, P. Fitzgerald, A. Tönjes, V. Gudnason, D. Toniolo, M. Pirastu, C. Bellenguez, R. S. Vasan, E. Ingelsson, A. L. Leutenegger, A. D. Johnson, A. L. DeStefano, S. Visvikis-Siest, S. Seshadri and M. Ciullo: Six Novel Loci Associated with Circulating VEGF Levels Identified by a Meta-analysis of Genome-Wide Association Studies. *PLoS Genet*, 12(2) (2016) doi:10.1371/journal.pgen.1005874

33. R. S. Sparkes, M. C. Sparkes, M. G. Wilson, J. W. Towner, W. Benedict, A. L. Murphree and J. J. Yunis: Regional assignment of genes for human esterase D and retinoblastoma to chromosome band 13q14. *Science*, 208(4447), 1042-4 (1980)

34. M. E. Askarian-Amiri, V. Seyfoddin, C. E. Smart, J. Wang, J. E. Kim, H. Hansji, B. C. Baguley, G. J. Finlay and E. Y. Leung: Emerging role of long non-coding RNA SOX2OT in SOX2 regulation in breast cancer. *PLoS One*, 9(7), e102140 (2014) doi:10.1371/journal.pone.0102140
